# A new species of *Nidirana* Dubois, 1992 (Anura, Ranidae) from Chongqing Municipality, China

**DOI:** 10.3897/BDJ.11.e101986

**Published:** 2023-05-10

**Authors:** Qi Ma, Zhijian Wang

**Affiliations:** 1 State Key Laboratory Breeding Base of Eco-Environment and Bio-Resource of the Three Gorges Area, School of Life Sciences, Southwest University, Chongqing, China State Key Laboratory Breeding Base of Eco-Environment and Bio-Resource of the Three Gorges Area, School of Life Sciences, Southwest University Chongqing China; 2 Chongqing Museum of Natural History, Chongqing, China Chongqing Museum of Natural History Chongqing China

**Keywords:** Chongqing Municipality, mitochondrial DNA, morphology, *Nidiranachongqingensis* sp. nov.

## Abstract

**Background:**

The *Nidirana* Dubois, 1992 exhibit a ubiquitous presence in East and Southeast Asia, spanning from Japan west to southern China and from northern Thailand to northern Vietnam and Laos in the south. The taxonomic categorisations pertaining to this genus continue to be a subject of debate, particularly with regard to those species that possess broad geographical distributions. In China, 18 species of Nidirana are currently recognised.

**New information:**

We describe a new species of this genus from south-western China. Mitochondrial 16S and COⅠ gene phylogenetic analyses support the new species as an independent clade nested within the *Nidirana*. The new species is phylogenetically close to *N.yaoica*, with a genetic distance of 2.5% from its sister taxon. Morphologically, the new species can be distinguished from congeners by a combination of the following characteristics: males relatively small (SVL 41.8-43.3 mm); lateroventral grooves present on both fingers and toes; relative finger length Ⅱ < Ⅰ < Ⅳ < Ⅲ; tibio-tarsal articulation reaching to the level of the eye or nostril; a pair of external subgular vocal sacs in males; one single nuptial pad on the dorsal surface of the base of the first finger in males during the breeding season; webbing formula I 1/2 - 1 II 1/2 - 2 III 1 - 2½ IV 2 - 1V.

## Introduction

The genus *Nidirana* Dubois, 1992 is found in subtropical and tropical parts of eastern and south-eastern Asia, ranging from Japan to the south of China, to the north of Thailand, Vietnam and Laos. The members of this genus typically inhabit natural or artificial swamps, ponds and paddy fields in hilly areas and, during the breeding season, some species construct nests (Fei et al. 2009, Grosjean et al. 2015, Lyu et al. 2017). The classification of *Nidirana* has been a subject of debate for a long time (Chen et al. 2005, Frost et al. 2006, Chuaynkern et al. 2010, Li et al. 2019, Lyu et al. 2019). It has been proposed as a subgenus or synonym of *Rana* Linnaeus, 1758 and merged with *Babina* Thompson, 1912 (Frost et al. 2006). However, based on molecular systematics, morphology and bioacoustics, Lyu et al. (2017) suggested that *Nidirana* is an independent genus and sister to *Babina*. In the past five years, several new species of this genus have been described, namely *N.leishanensis* Li, Wei, Xu, Cui, Fei, Jiang, Liu and Wang, 2019, *N.yaoica* Lyu, Mo, Wan, Li, Pang and Wang, 2019, *N.guangdongensis* Lyu, Wan and Wang, 2020, *N.mangveni* Lyu, Qi and Wang, 2020, *N.occidentalis* Lyu, Yang and Wang, 2020, *N.xiangica* Lyu and Wang, 2020, *N.yeae* Wei, Li, Liu, Cheng, Xu and Wang, 2020, *N.guangxiensis* Mo, Lyu, Huang, Liao and Wang, 2021, *N.guibeiensis* Chen, Ye, Peng and Li, 2022 and *N.shiwandashanensis* Chen, Peng, Li and Liu, 2022. (Li et al. 2019, Lyu et al. 2019, Lyu et al. 2020a, Lyu et al. 2020b, Wei et al. 2020, Lyu et al. 2021, Chen et al. 2022a, Chen et al. 2022b).

The conservative phenotype of many newly-described species has caused them to be misidentified as other congeneric species (Lyu et al. 2019, Lyu et al. 2020a, Lyu et al. 2021). As an example, Lyu et al. (2020b) identified three new species (*N.guangdongensis*, *N.mangveni* and *N.xiangica*) from multiple populations that were previously believed to be *N.adenopleura*. In addition, Chen et al. (2022a) described another new species, *N.shiwandashanensis*, from Shiwandashan National Nature Reserve in Shangsi County, Guangxi Province of China.

During our field survey in Qiangjiang District of Chongqing Municipality in southern China in 2022, we collected three specimens of frogs, which were identified as a species of *Nidirana*, based on the comparison of their morphological features. Upon further analysis of molecular systematics and morphology, these specimens were distinct from all known species belonging to the genus *Nidirana* and we herein describe them as a new species.

## Materials and methods

**Figure 1.**
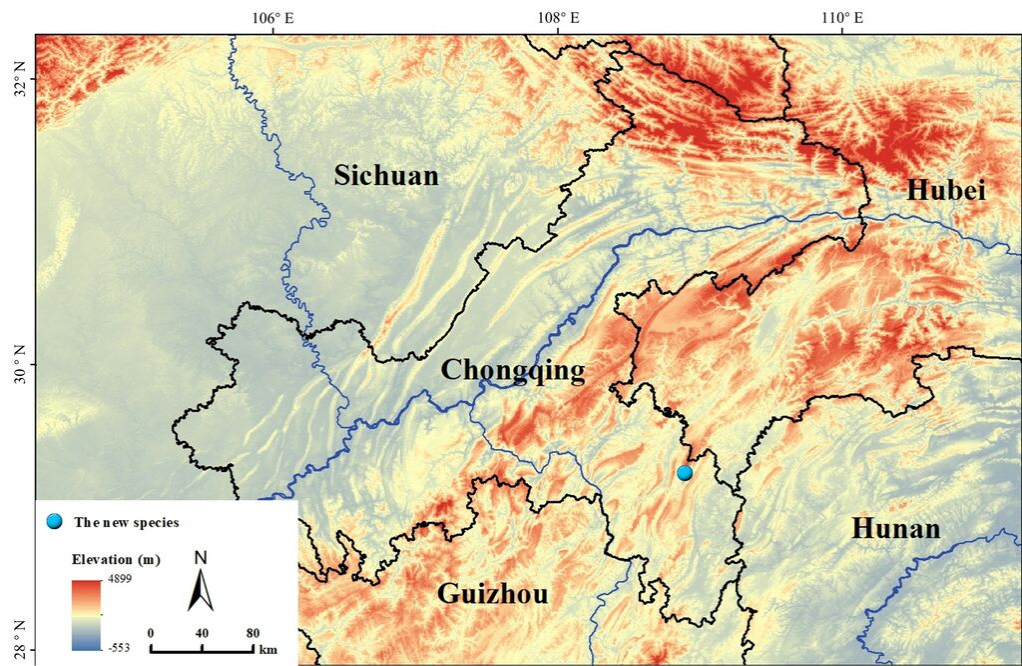
Geographical location of the type locality of the new species (blue dot).

Three adult male specimens of an undescribed species of *Nidirana* were collected from Qianjiang District, Chongqing Municipality, China in this study (see Fig. 1). In the field, the frog was euthanised with isoflurane and the specimens were fixed in 75% ethanol. Tissue samples were taken and preserved in 100% ethanol before fixation. The specimens were subsequently deposited in the Southwest University (SWU).

### Molecular analysis

Total DNA was extracted using the Tiangen DNA Extraction Kit DP304 (Tiangen Biochemical Technology Co., Ltd.), quantified for quantity and concentration using a spectrophotometer (Thermo Scientific NanoDrop 2000), and then kept at -20°C. Two fragments of the mitochondrial 16S rRNA (16S) and the cytochrome oxidase subunit I (COI) genes were amplified. Primers used for 16S were L3975 (5’-GCCTGTTTACCAAAAACAT-3’) and H4551 (5’-CCGGTCTGAACTCAGATCACGT-3’), and L2A (5’-CCAAACGAGCCTAGTGATAGCTGGTT-3’) and H10 (5’-TGATTACGCTACCTTTGCACGGT-3’), and for COI were dgLCO (5’-GGTCAACAAATCATAAAGAYATYGG-3’) and dgHCO (5’-AAACTTCAGGGTGACCAAARAAYCA-3’), following Lyu et al. (2019). The following steps were used to carry out a standard polymerase chain reaction (PCR) amplification in a 20 μl system: de-naturing step at 95°C for 4 min, 35 cycles of denaturing at 94°C for 40 s, annealing at 53°C (for 16S) / 48 °C (for COI) for 40 s and extending at 72°C for 60 s, and a final extending step at 72°C for 10 min. The PCR amplification products were detected by agarose gel electrophoresis. The products were then sent to Biotech Bioengineering (Shanghai) Co. All newly- obtained sequences have been submitted to GenBank (to be uploaded upon article acceptance). In order to investigate the current phylogenetic relationships amongst *Nidirana*, a dataset was constructed by concatenating 16S (~ 1102 bp) and COI (~ 562 bp) gene sequences.

From GenBank, we retrieved the homologous sequences of the related species in the genus *Nidirana*, as well as those of the outgroups *Babinaholsti* and *Babinasubaspera* (Kakehashi et al. 2013) (Table 1). Sequences that had been amplified recently were manually checked using SeqMan in the DNASTAR LASERGENE v.6 package. Sequences that had been sequenced in both directions were then combined, and the combined sequences were imported into MEGA 6 (Tamura et al. 2013) for comparison and the elimination of redundant sequences. In this study, phylogenetic trees were created using Bayesian Iinference (BI) and Mmaximum Llikelihood (ML) methods. ML was analyszed on the CIPRES Sscience Gateway with 100 rapid bootstrap replicates (Miller et al. 2010) (https://www.phylo.org/portal2). Bayesian methods were implemented in MrBayes v.3.2 (Ronquist et al. 2012) and the optimal substitution model was determined using ModelFinder (Kalyaanamoorthy et al. 2017), with the best model determined by ModelFinder in this article being GTR+I+G. In the Bayesian analysis, four Markov chains were used, operated for 10 million generations, sampled every 500 generations, and with the first 25% samples beingwere discarded as burn-in. The software Tracer v.1. 5 (Drummond and Rambaut 2007) was used to determine whether the resulting results converged (ESS > 200).

### Morphological characteristics

Measurements were taken with an electronic digital caliper (SHAHE brand, range 0-200 mm) with a measurement accuracy of 0.1 mm. The methods for measuring adult external morphological traits were according to Fei et al. (2009). The morphological characteristics are as follows: SVL, snout-vent length (the length from the snout to the posterior margin of the vent); HL, head length (the length from the snout to the articulation of the jaw); HW, head width (the widest distance between the two sides of the head); SL, snout length (the distance from the snout to the anterior corner of the eye); IND, internasal distance (minimum distance between the inner margins of the external nares); IOD, interorbital distance (the shortest distance between the inner edges of the upper eyelids); NSL, nostril-snout length (the length from the centre of the nostril to the tip of the snout); NEL, nostril-eye length (the length from the centre of the nostril to the anterior corner of the eye); UEW, upper eyelid width (margins measured perpendicular to the anterior-posterior axis); ED, eye diameter (distance from the anterior corner to the posterior corner of the eye); TD, tympanum diameter (horizontal diameter of tympanum); FAHL, forearm and hand length (the length from the elbow joint to the tip of finger IV); HAL, hand length (the length from proximal edge of inner palmar tubercle to the tip of the third finger); FAW, maximum width of forearm (the widest distance between the two sides of the forearm); HLL, hind limb length (the length from tip of fourth toe to vent); THL, thigh length (distance from vent to knee); TBL, tibia length (distance from knee to tarsus); TFL, length of tarsus and foot (the length from the tibiotarsal articulation to the tip of the toe IV); FTL, foot length (distance from distal end of shank to the tip of Toe IV).

Sex can be determined by examining the nuptial pad, vocal sac and suprabrachial gland, the webbing formula following Savage and Heyer (1997).

The unnamed species is also separated from other species of *Nidirana* based on morphological features. Morphological data for comparison were obtained from literature (Boettger 1895, Boulenger 1904, Boulenger 1909, Schmidt 1925, Chang and Hsü 1932, Bourret 1937, Kuramoto 1985, Chou 1999, Fei et al. 2007, Matsui 2007, Fei et al. 2009, Chuaynkern et al. 2010, Lyu et al. 2017, Li et al. 2019, Lyu et al. 2020a, Lyu et al. 2020b, Wei et al. 2020, Chen et al. 2022a, Chen et al. 2022b).

## Data resources

The accession numbers of the newly-discovered sequences in this analysis are displayed in Table 1 and all of the sequences in this study were obtained from GenBank.

## Taxon treatments

### Nidirana chongqingensis

Ma & Wang sp. nov.

1EFA6383-F50B-5F28-8FA8-EB169D904B02

B964B6E8-4D84-4D16-90FF-068E45BA6AB0

#### Materials

- **Type status:** Holotype. **Occurrence:** catalogNumber: SWU0001408; recordedBy: Qi Ma; individualCount: 1; sex: male; lifeStage: adult; occurrenceID: C3B1F1AD-5D4C-5F35-8F0C-7020A48D4D15; **Taxon:** scientificName: *Nidiranachongqingensis*; kingdom: Animalia; phylum: Chordata; class: Amphibia; order: Anura; family: Ranidae; genus: Nidirana; **Location:** higherGeography: South-western China; country: China; municipality: Chongqing; locality: Gaolu Village, Mala Town, Qianjiang District; verbatimElevation: 1419 m; verbatimCoordinates: 29°14'31.32"N 108°54'7.93"E; georeferenceSources: Google Earth; **Identification:** identifiedBy: Qi Ma; **Event:** eventDate: 03/07/2022; **Record Level:** basisOfRecord: PreservedSpecimen
- **Type status:** Paratype. **Occurrence:** catalogNumber: SWU0001439; recordedBy: Qi Ma; individualCount: 2; sex: male; lifeStage: adult; occurrenceID: A44D36F5-8AD4-5B9E-A834-324FA55C768E; **Taxon:** scientificName: *Nidiranachongqingensis*; kingdom: Animalia; phylum: Chordata; class: Amphibia; order: Anura; family: Ranidae; genus: Nidirana; **Location:** higherGeography: South-western China; country: China; municipality: Chongqing; locality: Gaolu Village, Mala Town, Qianjiang District; verbatimElevation: 1419 m; verbatimCoordinates: 29°14'31.32"N 108°54'7.93"E; georeferenceSources: Google Earth; **Identification:** identifiedBy: Qi Ma; **Event:** eventDate: 03/07/2022; **Record Level:** basisOfRecord: PreservedSpecimen

#### Diagnosis

The new species was placed in the genus *Nidirana* based on molecular data (16S and COI genes).

The new species could be distinguished from its congeners by a combination of the following characters: (1) body size small (SVL 41. 8-43. 3 mm in males); (2) indistinct canthus rostralis; (3) no longitudinal ridges on the upper arm; (4) lateroventral grooves on the ventral side of all fingers and toes; (5) supernumerary tubercles below the base of fingers III and IV, inconspicuous; (6) metacarpal tubercles distinct and prominent; (7) well-developed dorsolateral folds, but intermittent posteriorly; (8) supratympanic fold absent; (9) males with large, smooth and protruding suprabrachial glands in breeding period; (10) dorsal skin relatively smooth, without horny spines on the back; (11) mid-dorsal stripe present; (12) ventral surface of body milky white, but the throat, chest and ventral side of the limbs are densely covered with brownish-red or brown-black spots; (13) tibio-tarsal articulation reaching the angle of the eye or the nostril when adpressed along body, heels not meeting or just meeting when hind limbs flexed at a straight angle to the body's axis; (14) a pair of external subgular vocal sacs in males; (15) males with one single nuptial pad on the dorsal surface first finger in the breeding period; (16) finger tips not dilated, tip of each toe slightly dilated (Table 2).

##### Etymology

The specific name "Chongqing" refers to the type locality of the new species in Chongqing Municipality, where the new species was collected.

##### Common name

“Chongqing Music Frog” in English and “重庆琴蛙 (Chóng qìng qín wā)” in Chinese.

##### Description of holotype

An adult male, SVL 43.3 mm, head longer than width (HL: HW = 1.18), flat above; snout rounded in dorsal view, slightly protruding beyond lower jaw; loreal region slightly inclined outwards, slightly concave in the middle; canthus rostralis indistinct; pupil elliptical, horizontal; nostril rounded, directed laterally, closer to the eye than to the snout; internasal distance larger than interorbital distance (IND:IOD = 1.18); from below anterior corner of the eye and tympanum to axial region, forming a maxillary gland in posterior corner of mouth, with a soybean size gland behind; tympanum distinct, tympanum diameter smaller than eye diameter (TD/ED=0.73); supratympanic fold absent; the presence of vomerine ridge, small teeth on the surface; a pair of external subgular vocal sacs present at the corners of the throat; tongue cordiform, deeply notched posteriorly.

Forelimbs moderately robust, with forearm and hand length being half of the body length (FAHL:SVL = 0.42); a large and smooth suprabrachial gland present behind the base of the forelimb, prominent; absent longitudinal ridges on the upper arm; tips of fingers rounded, not dilated; lateroventral grooves of fingers meeting at the disc's tip; fingers thin, free of webbing, with narrow lateral fringes; subarticular tubercles clearly visible, rounded and with a distinct protuberance; supernumerary tubercles below the base of finger present on fingers III and IV; relative length of fingers: II < I < IV < III; palmar tubercles three, which are long and elliptic.

Hind limbs relatively robust, tibia length 55% of SVL and foot length 52% of SVL; heels not meeting or just meeting when hind limbs flexed at right angles to axis of body; tibio-tarsal articulation reaching the eye angle or nostril when hind limb is stretched along the side of the body; several longitudinal ridges on the back of the thigh and tibia; thigh length shorter than tibia length (THL:TBL = 0.89), length of tarsus and foot longer than tibia length (TFL:TBL = 1.37); toes relatively long and thin, relative lengths I < II < V < III < IV; webbing moderate, webbing formula: I 1/2 - 1 II 1/2 - 2 III 1 - 2½ IV 2 - 1V; toes with lateral fringes; prominent and rounded subarticular tubercles; inner metatarsal tubercles oval, outer metatarsal tubercles small and rounded.

Dorsal anterior region of skin relatively smooth; several large warts on the middle and posterior back; posterior dorsum of body rough with small protrusions, but no horny spinules on them; dorsolateral fold relatively thin and distinct, extending from posterior corner of the eye to above of groin, but discontinuous in the posterior; a noticeable, large, smooth suprabrachial gland behind the base of the forelimb; ventral skin smooth, small tubercles around the region close to dorsolateral fold; body side and limbs relatively smooth; dorsum of tibia relatively rough, forming several longitudinal ridges; ventral skin of the body is smooth, flattened tubercles densely distributed on the rear of thigh and around vent.

##### Colouration of holotype in life

**Figure 2.**
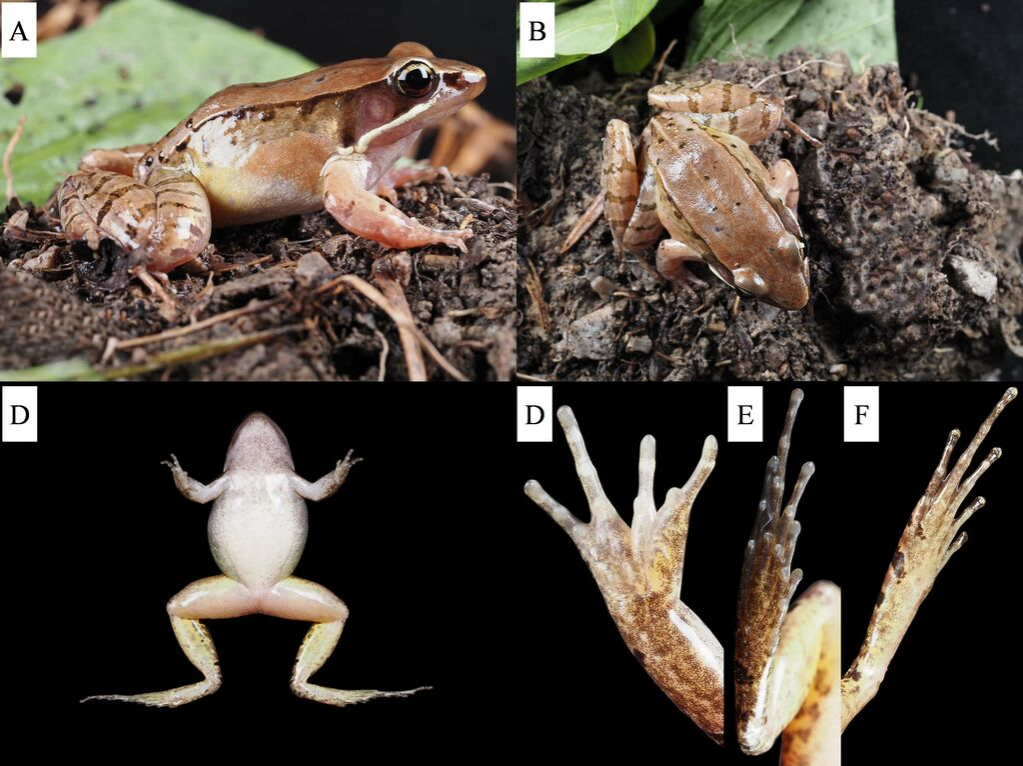
Holotype of *Nidiranachongqingensis* sp. nov. (SWU0001408) in life. **A** lateral view; **B** dorsal view; **C** ventral view; **D** ventral view of hand; **E** ventral view of foot; **F** dorsal view of foot.

The colouration of the dorsal skin varies, mostly being brown, several tubercles on the flanks and posterior region, some of which have a black spot. A light brown mid-dorsal stripe starts from the mid-dorsal region and begins at the eye angle area, extending backwards to vent, becoming more distinct posteriorly, without dark brown edges; dorsolateral folds brown on the upper part and dark brown on the lower part; flanks cream-yellow on the upper part and light grey on the lower part; limbs brown with black-brown transverse bars; loreal and temporal regions dark brown; tympanum red-brown; upper 1/3 iris bright yellow and lower 2/3 brown-red; ventral surface of body milky white; ventral surfaces of throat, anterior chest, upper limbs and flank of trunk densely covered with brown-red or brown-black spots, while spots on lower limbs relatively sparse; maxillary gland yellow-white; ventral side of the forelimbs milky white; upper part of the thigh ventral side light yellow, lower part flesh red; ventral side of the tibia light yellow (Fig. 2).

##### Colouration of holotype in preservation

**Figure 3.**
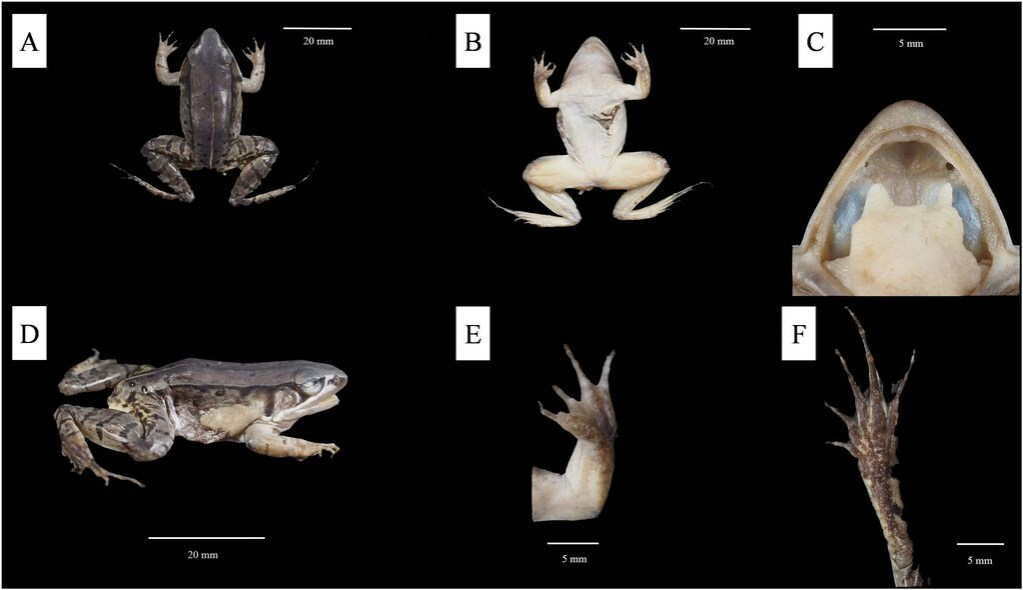
Holotype of *Nidiranachongqingensis* sp. nov. (SWU0001408) in preservative. **A** dorsal view; **B** ventral view; **C** view of oral cavity; **D** lateral view; **E** ventral view of hand; **F** ventral view of foot.

Colouration of dorsal surface fading to blackish-brown; black spots on dorsum and flank more distinct; upper limbs fading to grey-white and lower limbs fading to brownish; transverse bars on the limbs brownish-black; ventral surface of body fading to grey-white; ventral surface of thigh buff-coloured (Fig. 3).

##### Variation

**Figure 4.**
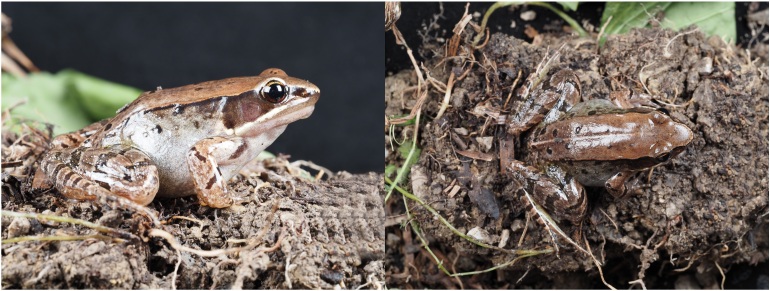
Colouration variation of the male paratype of *Nidiranachongqingensis* sp. nov. (SWU0001435) in life. **A** lateral view; **B** dorsal view.

Measurements of the type specimens are listed in Table 2. Paratypes are similar to the holotype in morphology and colouration. The dorsal side colouration is light brown or dark brown; a light brown mid-dorsal stripe starts from the area between the eyes and extends backwards to vent and becomes more obvious; ventral side colouration milky white or grey-white; transverse stripes on the limbs light brown or dark brown; black spots on the back sparser and darker (Fig. 4).

##### Secondary sexual characters

A pair of external subgular vocal sacs present in males; nuptial pad on the inner side of base of fingers Ⅰ; nuptial spinules invisible; the presence of suprabrachial gland.

##### Distribution and habitats

*Nidiranachongqingensis* sp. nov. is known from the type locality in Qianjiang District, Chongqing Municipality, China, at elevations between 1400 and 1500 metres above sea level.

#### Ecology

**Figure 5.**
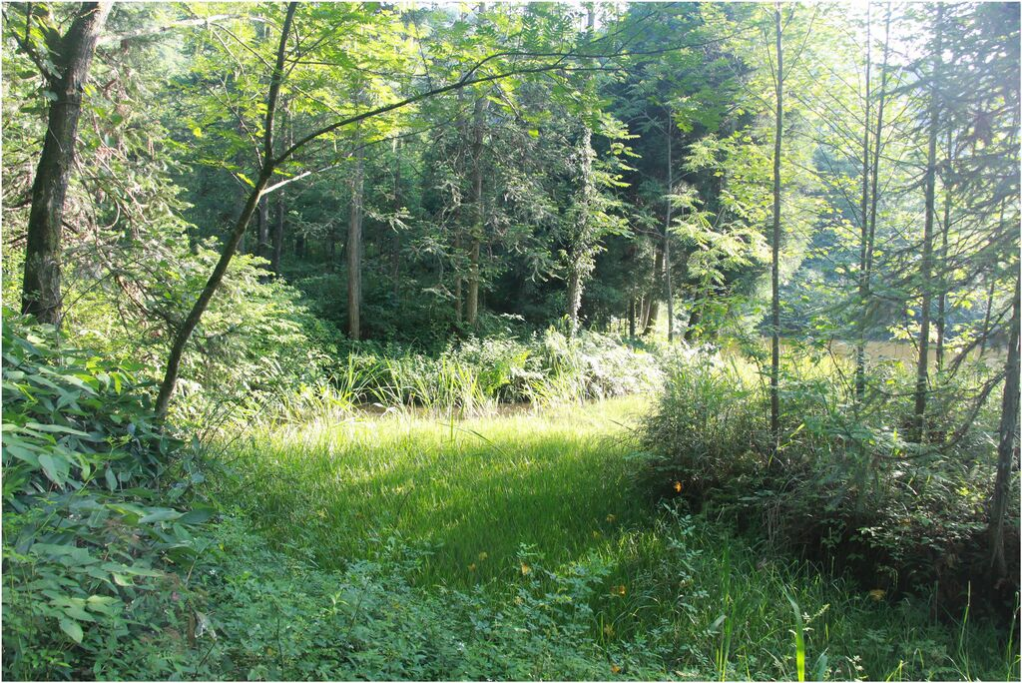
Habitat of *N.chongqingensis* sp. nov. in the type locality, Qianjiang District, Chongqing Municipality, China.

*Nidiranachongqingensis* sp. nov. inhabits mountain swamps, ponds, aquatic grassland and nearby weed thickets (Fig. 5). Due to the dense weeds, the frog is hard to find while it is hiding in them. The males were seen to call at night.

## Analysis

### Phylogenetic

**Figure 6.**
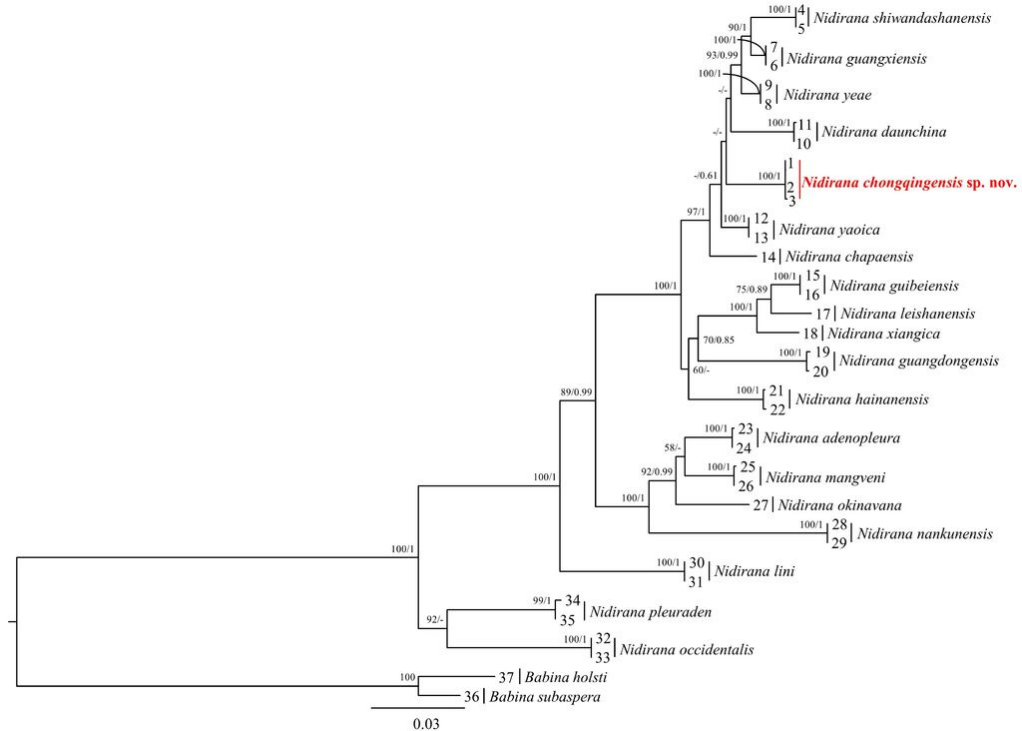
Phylogenetic tree reconstructed using Maximum Likelihood (ML) and Bayesian Inference (BI) methods, based on mitochondrial 16S + COI genes. Numbers denote the Maximum Likelihood bootstrap values (left) and Bayesian posterior probabilities (right). Samples 1–37 refer to Table 1. The symbol “–” represents value below 50/0.6.

The aligned sequence matrix of 16S + COI comprised 1664 bp. Our ML and BI analyses produced nearly identical phylogenetic topologies, which was consistent with the result of Chen et al. (2022b). The phylogenetic results showed that the *Nidirana* genus included two species groups that could be further divided into four highly-supported clades, denoted as A, B, C and D (following Lyu et al. (2017)) (Fig. 6). The *Nidirana* populations from Qianjiang, Chongqing (ID 1-3) comprising of three specimens were assigned to Clade C of the *N.adenopleura* group and were distant from true *N.adenopleura* in Clade D in the phylogeny. Uncorrected *p*-distances amongst all *Nidirana* species used in this study are presented in Table 3.

The new species is embeded in the same clade with *N.shiwandashanensis*, *N.guangxiensis*, *N.yeae*, *N.daunchina*, *N.yaoica* and *N.chapaensis*. The relationships amongst these seven lineages remain unresolved, although the Qianjiang, Chongqing population appears to be more closely related to *N.yaoica*.

For the COI gene, the genetic distance between the new species and the closely-related species *N.yaoica* was 2.5%, respectively, approximately the same level as the distance between *N.guangxiensis* and *N.yeae* (1.8%), *N.yaoica* and *N.yeae* (2.3%) (see Table 3).

Molecular phylogenetic analyses revealed that the *Nidirana* population from Qianjiang District, Chongqing Municipality, China is distinct from its congeners.

### Morphology

A summary of morphological characters is provided in Suppl. material 1. *Nidiranachongqingensis* sp. nov. differs from its congeners in the following characteristics: (1) small-sized body, SVL 41.8-43.3 mm in adult males [vs. SVL < 38 mm in adult male *N.nankunensis*; SVL > 46.2 mm in adult male *N.shiwandashanensis*, *N.guangdongensis*, *N.guibeiensis*; *N.leishanensis*, *N.mangveni*, *N.pleuraden* and *N.xiangica*]; (2) relative length of fingers: II < I < IV < III [vs. Ⅰ < Ⅱ < Ⅳ < Ⅲ in *N.mangveni*; Ⅱ < Ⅰ = Ⅳ < Ⅲ in *N.chapaensis*; Ⅱ < Ⅳ < Ⅰ < Ⅲ in *N.yeae*, *N.shiwandashanensis* and *N.leishanensis*; II < IV < I < III in *N.guibeiensis*]; (3) lateroventral grooves present on all fingers [vs. absent on all fingers in *N.yeae*, *N.occidentalis* and *N.pleuraden*; absent or rarely present in *N.daunchina*; present on all fingers, except finger I in *N.nankunensis*, *N.chapaensis*, *N.okinavana*, *N.adenopleura*, *N.lini*, *N.guangdongensis* and *N.guibeiensis*; present on fingers Ⅲ and IV in *N.guangxiensis* and *N.mangveni*]; (4) lateroventral grooves present on all toes [vs. absent on all toes in *N.occidentalis* and *N.pleuraden*]; (5) tibio-tarsal articulation reaches anterior corner of the eye or nostril [vs. beyond the snout in *N.lini*; at the eye in *N.yeae* and *N.occidentalis*]; (6) dorsal skin relatively smooth, without horny spines on the back [vs. white horny spinules on the entirely dorsum, dorsolateral folds, flanks and dorsal hind limbs in *N.guangdongensis*; white horny spinules on the entirely dorsum, dorsolateral folds, flanks, dorsal limbs, loreal region and temporal region including tympanum in males in *N.xiangica*, white horny spinules on the posterior or entire dorsum in males in *N.mangveni*; present in *N.adenopleura*, *N.lini* and *N.pleuraden*]; (7) during the breeding season, nuptial pads present on the first finger [vs. no nuptial pad is present in *N.hainanensis*; the nuptial pad of the first finger is divided into two parts in *N.chapaensis*; nuptial pads present on the first and second finger respectively in *N.leishanensis*]; (8) males possess a pair of external subgular vocal sacs in males [vs. absent in *N.okinavana*]; (9) finger tips not dilated [vs. dilated in *N.shiwandashanensis*, *N.adenopleura*, *N.chapaensis*, *N.daunchina*, *N.guangdongensis*, *N.guangxiensis*, *N.guibeiensis*, *N.hainanensis*, *N.leishanensis*, *N.lini*, *N.mangveni*, *N.nankunensis*, *N.okinavana*, *N.xiangica*, *N.yaoica* and *N.yeae*]; (10) tip of each toe dilated [vs. not dilated in *N.occidentalis* and *N.pleuraden*]; (11) supratympanic fold absent [vs. weak supratympanic fold present in *N.mangveni*]; (12) heels not meeting or just meeting when hind limbs flexed at right angles to axis of body [vs. heels overlapping in *N.adenopleura*, *N.guangdongensis*, *N.guangxiensis*, *N.leishanensis*, *N.lini*, *N.mangveni*, *N.nankunensis*, *N.okinavana* and *N.yaoica*]; (13) indistinct canthus rostralis [vs. distinct in *N.adenopleura*, *N.daunchina*, *N.guangdongensis*, *N.guangxiensis*, *N.hainanensis*, *N.lini*, *N.mangveni*, *N.shiwandashanensis* and *N.xiangica*]; (14) no longitudinal ridges on upper arms [vs. weak longitudinal ridges on upper arms and slightly extending to lower arm in *N.guangxiensis*]; (15) supernumerary tubercles below the base of finger III and IV, inconspicuous [vs. distinct in *N.guangxiensis*, *N.mangveni* and *N.xiangica*; absent in *N.okinavana*]; (16) dorsal skin relatively smooth [vs. rough with dense granules in *N.guangxiensis*].

Phylogenetically, *N.chongqingensis* sp. nov. is closest to *N.yaoica*. However, *N.chongqingensis* sp. nov. differs from *N.yaoica* in the following characteristics: (1) indistinct canthus rostralis [vs. distinct in *N.yaoica*]; (2) maxillary gland from below anterior corner of the eye [vs. from below nostril in *N.yaoica*]; (3) the absence of longitudinal ridges on the upper arm [vs. present in *N.yaoica*]; (4) finger tips not dilated, rounded [vs. discs of digits dilated, pointed]; (5) lateroventral grooves meeting at the disc's tip [vs. not meeting in *N.yaoica*]; (6) heels not meeting or just meeting when hind limbs flexed at right angles to axis of body [vs. heels overlapping in *N.yaoica*]; (7) absent wide dark brown edges of the mid-dorsal strip [vs. dark brown edges of the mid-dorsal stripe more distinct in *N.yaoica*].

## Discussion

To date, within the confines of Chongqing Municipality, a duo of acknowledged organisms of the genus *Nidirana* have been identified, namely *N.adenopleura* and *N.daunchina*. Owing to their cautious phenotypic traits, the *Nidirana* species located in the southern region of China typically fall under the classification of *N.adenopleura* (Lyu et al. 2017, Lyu et al. 2020a, Lyu et al. 2020b). The distribution range of *N.adenopleura* is deemed to be the most extensive, encompassing Iriomote and Ishigaki Islands, as well as the Yaeyama group of Ryukyu Island in Japan, Taiwan and several Provinces in China, such as Guizhou, Anhui, Zhejiang, Jiangxi, Hunan, Fujian, Guangdong and Guangxi (Fei et al. 2009, Fei et al. 2012, Li et al. 2019, Frost 2023). As stated by Fei et al. (2009), the survival of this particular species is heavily reliant on stillwater habitats. Given its extensive range of distribution and specific habitat demands, it is presumed that population divergence and potentially even species divergence, could be facilitated. Our newly-discovered species *N.chongqingensis* sp. nov. indicate that previous studies, which only regarded the *Nidirana* population from Chongqing Municipality as *N.adenopleura*, need further verification.

## Supplementary Material

XML Treatment for Nidirana chongqingensis

8F08A3B5-0C31-59CA-A227-24BB696BBF16 10.3897/BDJ.11.e101986.suppl1 Supplementary material 1 Diagnostic characters separating *Nidiranachongqingensis* sp. nov. from all congeners Data type morphological File: oo_840753.xlsx https://binary.pensoft.net/file/840753 Qi Ma, Zhijian Wang

## Figures and Tables

**Table 1.** Localities, voucher information and GenBank numbers for samples used in this study. An asterisk denotes type localities.

ID	Species	Locality (* type locality)	Voucher No.	16S	COI	References
1	*N.chongqingensis* sp. nov.	China: Chongqing: Qianjiang District	SWU0001408	OQ846777	OQ843905	This study
2	*N.chongqingensis* sp. nov.	China: Chongqing: Qianjiang District	SWU0001439	OQ846779	OQ843907	This study
3	*N.chongqingensis* sp. nov.	China: Chongqing: Qianjiang District	SWU0001435	OQ846778	OQ843906	This study
4	*N.shiwandashanensis*	China: Guangxi: Shangsi County	NNU00238	MZ787977	MZ782098	Chen et al. (2022a)
5	*N.shiwandashanensis*	China: Guangxi: Shangsi County	NNU00239	MZ787978	MZ782099	Chen et al. (2022a)
6	*N.guangxiensis*	China: Guangxi: Mt Daming*	NHMG 202007001	MZ677222	MZ678729	Lyu et al. (2021)
7	*N.guangxiensis*	China: Guangxi: Mt Daming*	NHMG 202007002	MZ677223	MZ678730	Lyu et al. (2021)
8	*N.yeae*	China: Guizhou: Tongzi County	CIB TZ20190608005	MN295228	MN295234	Wei et al. (2020)
9	*N.yeae*	China: Guizhou: Tongzi County	CIB TZ20160714016	MN295231	MN295237	Wei et al. (2020)
10	*N.daunchina*	China: Sichuan: Mt Emei*	SYS a004594	MF807822	MF807861	Lyu et al. (2020a)
11	*N.daunchina*	China: Sichuan: Mt Emei*	SYS a004595	MF807823	MF807862	Lyu et al. (2020a)
12	*N.yaoica*	China: Guangxi: Mt Dayao*	SYS a007020	MK882276	MK895041	Lyu et al. (2019)
13	*N.yaoica*	China: Guangxi: Mt Dayao*	SYS a007021	MK882277	MK895042	Lyu et al. (2019)
14	*N.chapaensis*	Vietnam: Lao Cai: Sapa*	MNHN 2000.4850	KR827711	KR087625	Grosjean et al. (2015)
15	*N.guibeiensis*	China: Guangxi: Xing’an: Maoershan	NNU 00917	ON985180	ON968962	Chen et al. (2022a)
16	*N.guibeiensis*	China: Guangxi: Xing’an: Maoershan	NNU 00918	ON985181	ON968963	Chen et al. (2022a)
17	*N.leishanensis*	China: Guizhou: Mt Leigong*	SYS a007908	MN946453	MN945209	Lyu et al. (2021)
18	*N.xiangica*	China: Hunan: Mt Dawei*	SYS a006492	MN946434	MN945190	Lyu et al. (2020a)
19	*N.guangdongensis*	China: Guangdong: Yingde City*	SYS a005767	MN946406	MN945162	Lyu et al. (2020a)
20	*N.guangdongensis*	China: Guangdong: Yingde City*	SYS a005768	MN946407	MN945163	Lyu et al. (2020a)
21	*N.hainanensis*	China: Hainan: Mt Diaoluo*	SYS a007669	MN946451	MN945207	Lyu et al. (2020a)
22	*N.hainanensis*	China: Hainan: Mt Diaoluo*	SYS a007670	MN946452	MN945208	Lyu et al. (2020a)
23	*N.adenopleura*	China: Taiwan: Taichung City	SYS a007358	MN946445	MN945201	Lyu et al. (2020a)
24	*N.adenopleura*	China: Taiwan: Taichung City	SYS a007359	MN946446	MN945202	Lyu et al. (2020a)
25	*N.mangveni*	China: Zhejiang: Mt Dapan*	SYS a006310	MN946424	MN945180	Lyu et al. (2020a)
26	*N.mangveni*	China: Zhejiang: Mt Dapan*	SYS a006311	MN946425	MN945181	Lyu et al. (2020a)
27	*N.okinavana*	Japan: Okinawa: Iriomote Island*	Not given	NC022872	NC022872	Kakehashi et al. (2013)
28	*N.nankunensis*	China: Guangdong: Mt Nankun*	SYS a005718	MF807839	MF807878	Lyu et al. (2017)
29	*N.nankunensis*	China: Guangdong: Mt Nankun*	SYS a005719	MF807840	MF807879	Lyu et al. (2017)
30	*N.lini*	China: Yunnan: Jiangcheng County*	SYS a003967	MF807818	MF807857	Lyu et al. (2017)
31	*N.lini*	China: Yunnan: Jiangcheng County*	SYS a003968	MF807819	MF807858	Lyu et al. (2017)
32	*N.occidentalis*	China: Yunnan: Mt Gaoligong*	SYS a003775	MF807816	MF807855	Lyu et al. (2020a)
33	*N.occidentalis*	China: Yunnan: Mt Gaoligong*	SYS a003776	MF807817	MF807856	Lyu et al. (2020a)
34	*N.pleuraden*	China: Yunnan: Kunming City*	SYS a007858	MT935683	MT932858	Lyu et al. (2020b)
35	*N.pleuraden*	China: Yunnan: Wenshan City	SYS a007717	MT935671	MT932850	Lyu et al. (2020b)
36	*Babinasubaspera*	Japan: Kagoshima: Amami Island*	Unknown	NC022871	NC022871	Kakehashi et al. (2013)
37	*Babinaholsti*	Japan: Okinawa*	Unknown	NC022870	NC022870	Kakehashi et al. (2013)

**Table 2.** Measurements of the new species (in mm). Abbreviations defined in Materials and Methods.

Measurement	SWU0001408 (Male, holotype)	SWU0001435 (Male, paratype)	SWU0001439 (Male, paratype)
SVL	43. 3	42. 0	41. 8
HL	17. 4	17. 3	16. 2
HW	14. 7	13. 4	13. 3
SL	7. 2	6. 5	6. 8
IND	5. 8	5. 5	5. 0
IOD	4. 9	4. 6	3. 2
NSL	3. 8	3. 4	3. 7
NEL	3. 7	3. 2	3. 0
UEW	4. 1	3. 9	3. 3
ED	5. 5	5. 3	5. 2
TD	4. 0	3. 1	3. 4
FAHL	18. 0	18. 1	17. 9
HAL	10. 7	11. 1	11. 4
FAW	3. 8	3. 5	2. 8
HLL	70. 1	67. 8	66. 5
THL	22. 6	20. 2	20. 4
TBL	23. 8	22. 5	22. 8
TFL	32. 5	31. 9	31. 2
FTL	22. 6	22. 2	22. 2

**Table 3.** Uncorrected *p*-distances based on COI genes amongst all Nidirana species (in 0.1%)

		1	2	3	4	5	6	7	8	9	10	11	12	13	14	15	16	17	18
1	*N.chongqingensis* sp. nov.																		
2	*N.shiwandashanensis*	3.9%																	
3	*N.guangxiensis*	4.1%	2.7%																
4	*N.yeae*	3.4%	2.7%	1.8%															
5	*N.daunchina*	4.8%	4.8%	4.3%	3.9%														
6	*N.yaoica*	2.5%	3.9%	3.0%	2.3%	3.7%													
7	*N.chapaensis*	3.2%	4.3%	3.4%	3.0%	4.8%	2.8%												
8	*N.guibeiensis*	5.9%	6.4%	5.9%	5.5%	6.2%	5.5%	5.5%											
9	*N.leishanensis*	5.5%	6.0%	5.5%	5.9%	6.2%	5.5%	5.5%	2.7%										
10	*N.xiangica*	6.0%	7.3%	6.0%	5.7%	6.4%	5.7%	5.3%	2.8%	3.0%									
11	*N.guangdongensis*	6.4%	6.8%	6.6%	5.2%	5.9%	5.3%	6.0%	5.7%	6.2%	6.4%								
12	*N.hainanensis*	4.3%	5.3%	5.2%	4.4%	5.3%	4.3%	4.3%	6.0%	5.5%	5.7%	5.7%							
13	*N.adenopleura*	8.9%	9.1%	8.5%	8.2%	8.9%	7.5%	8.2%	9.1%	9.6%	10.5%	8.9%	8.4%						
14	*N.mangveni*	9.8%	10.3%	10.0%	9.4%	9.6%	9.1%	9.3%	10.1%	10.7%	10.9%	9.8%	9.4%	4.8%					
15	*N.nankunensis*	10.0%	11.2%	10.3%	9.6%	10.9%	10.0%	9.6%	9.8%	9.8%	10.1%	9.1%	10.5%	7.1%	6.9%				
16	*N.lini*	9.4%	9.6%	9.6%	8.5%	9.3%	9.6%	8.5%	10.1%	11.0%	10.9%	9.6%	9.4%	9.6%	9.8%	9.3%			
17	*N.occidentalis*	12.3%	11.4%	12.5%	12.1%	11.2%	11.7%	11.0%	12.8%	13.2%	12.8%	12.6%	11.7%	11.2%	11.2%	12.5%	11.4%		
18	*N.pleuraden*	12.6%	11.7%	12.6%	11.6%	11.9%	12.1%	12.3%	14.2%	14.2%	14.1%	11.7%	11.7%	11.0%	12.8%	11.7%	10.9%	8.4%	
19	*N.okinavana*	10.1%	9.6%	8.9%	8.9%	9.4%	8.9%	8.5%	10.1%	10.7%	11.0%	9.3%	9.4%	4.6%	4.8%	7.1%	10.3%	11.4%	11.2%
